# Random guess and wishful thinking are the best blinding scenarios

**DOI:** 10.1016/j.conctc.2016.05.003

**Published:** 2016-05-07

**Authors:** Heejung Bang

**Affiliations:** Division of Biostatistics, Department of Public Health Sciences, University of California, Davis, CA, USA

**Keywords:** Bias, Blinding, Blinding index, Clinical trial

## Abstract

Blinding is a methodologic safeguard of treatment evaluation, yet severely understudied empirically. Mathieu et al.'s theoretical analysis (2014) provided an important message that blinding cannot eliminate potential for bias associated with belief about allocation in randomized controlled trial; just like the intent-to-treat principle does not guarantee unbiased estimation under noncompliance, the blinded randomized trial as a golden standard may produce bias. They showed possible biases but did not assess how large the bias could be in different scenarios. In this paper, we examined their findings, and numerically assessed and compared the bias in treatment effect parameters by simulation under frequently encountered blinding scenarios, aiming to identify the most ideal blinding scenarios in practice. We conclude that Random Guess and Wishful Thinking (e.g., participants tend to believe they received treatment) are the most ideal blinding scenarios, incurring minimal bias. We also find some evidence that imperfect or partial blinding can be better than no blinding.

## 1 Introduction

Blinding is a critical feature in comparative evaluations to minimize various biases. Blinded randomized controlled trial (RCT) is widely accepted as a gold standard when we compare treatments, whenever feasible [1], [2], [3], [4], [5]. Blinding can be more relevant and important to subjective outcomes, such as patient-reported outcomes. Several authors reviewed the current practice of blinding-related techniques, assessment and reporting [2], [6], and it has been suggested that unblinding may overestimate the treatment effect [7], [8]. Although the role and importance of blinding are well recognized in the clinical trial community, statistical investigation on this topic has been rare, partly due to inherently complicated and subjective/qualitative nature.

Recently, Mathieu et al. (2014) provided a theoretical analysis demonstrating that blinding cannot eliminate potential for bias associated with belief about allocation in RCT, which could be surprising or counterintuitive for many trialists [1], [9]. Specifically, they studied a mathematical framework of simple RCTs, and identified conditions where the bias in treatment effect is equal to zero. Except for highly restrictive conditions, if belief about the treatment allocation is translated into the study outcome (e.g., over or under-reporting of the outcome), the bias is expected to be non-zero. Thus, the authors concluded that blinding cannot guarantee to prevent bias caused by belief, but emphasized that it is not their intention to suggest that RCTs should not be blinded. They considered deterministic/hypothetical scenarios under a type of effective blinding, without numerical evaluation.

In this paper, we intend to study Mathieu et al.'s findings carefully and assess the bias in different treatment effect parameters numerically in more practical/realistic settings, under qualitatively different blinding scenarios, with a goal to provide a better insight and some actionable advice for trialists, if any. In section 2, we present background and mathematical framework. In section 3, we perform simulation studies and summarize the findings. Discussions and conclusions are provided at the end.

## 2 Mathematical framework

We summarize a simple, theoretical framework posited by Mathieu et al. as basis, examine and adapt here [9]. Each cell in a 3 × 2 table for guess status by allocation has the number of subjects n_ij_, where j denotes allocation (T = treatment, C = control) and i denotes belief about allocation (t = treatment, u = don't know, c = control); see Table 1. We assume that outcomes can be distorted via two mechanisms, where a_i_ is the magnitude of distortion that is independent of the true outcome Y_ij_ (e.g., fixed, *a priori* expectation before allocation) and b_i_ is that proportional to the outcome (e.g., unblinding during trial) in both arms. Thus, the total distortion or bias in the individual outcome due to belief in a cell is a_i_ + b_i_Y_ij_; the observed outcome is

$$Y_{ij}^{\prime }=Y_{ij}+a_{i}+b_{i}Y_{ij}\text{.}$$

**Table 1:** Statistical notation.

Treatment	Allocation (*j*), outcome & sample size			
Guess (*i*)	Treatment arm (T)		Control arm (C)	
Received treatment (t)	$Y_{tT}^{\prime }=Y_{tT}+a_{t}+b_{t}Y_{tT}$	*n*_*tT*_	$Y_{tC}^{\prime }=Y_{tC}+a_{t}+b_{t}Y_{tC}$	*n*_*tC*_
Don't know (u)	$Y_{uT}^{\prime }=Y_{uT}+a_{u}+b_{u}Y_{uT}$	*n*_*uT*_	$Y_{uC}^{\prime }=Y_{uC}+a_{u}+b_{u}Y_{uC}$	*n*_*uC*_
Received control (c)	$Y_{cT}^{\prime }=Y_{cT}+a_{c}+b_{c}Y_{cT}$	*n*_*cT*_	$Y_{cC}^{\prime }=Y_{cC}+a_{c}+b_{c}Y_{cC}$	*n*_*cC*_

See Table 1 in Mathieu et al. (2014).

If there is no distortion, then a_i_ = b_i_ = 0 so ${\text{Y}}_{\text{ij}}^{\prime }={\text{Y}}_{\text{ij}}$, i.e., individual outcome is unbiased.

We consider three treatment effect parameters: mean difference, mean ratio and odds ratio (OR) where OR is relevant when the outcome is binary. For binary outcome, mean difference and ratio are typically interpreted as incidence or risk difference (RD) and risk ratio (RR) in epidemiologic and clinical trial contexts.

Let us assume the randomization of the 1:1 allocation ratio for two treatments, and define the n_ij_-weighted mean of all ${\text{Y}}_{\text{ij}}^{\prime }$ as $\sum_{i}\left(n_{ij}Y_{ij}^{\prime }\right)/\sum_{i}n_{ij}$ for j = T and C. If belief about allocation is independent of actual allocation, so n_iT_ = n_iC_ = n_i_, which can be a specific form of effective blinding (see its connection to the ‘Wishful thinking’ scenario below) [9], then the absolute estimate of the treatment effect is unbiased:

$$\sum_{i}\left(n_{iT}Y_{iT}^{\prime }\right)/\sum_{i}n_{iT}-\sum_{i}\left(n_{iC}Y_{iC}^{\prime }\right)/\sum_{i}n_{iC}=\sum_{i}\left(n_{iT}Y_{iT}\right)/\sum_{i}n_{iT}-\sum_{i}\left(n_{iC}Y_{iC}\right)/\sum_{i}n_{iC}$$

if, but not only if, in every stratum, either (1) b_i_ = 0 or (2) Y_iC_ = Y_iT_ (i.e., there is no effect of treatment). We can further show that when n_iT_ = n_iC_ = n_i_ may or may not be true, if (3) $\;\sum_{i}n_{iT}a_{i}\;=0$, $\sum_{i}n_{iT}b_{i}\;Y_{iT}=0$, $\sum_{i}n_{iC}a_{i}=0$, and $\sum_{i}n_{iC}b_{i}\;Y_{iC}=0$, then unbiased estimation is achieved. Here, the conditions in (3) hold, for example, if n_tT_ = n_cT_, n_tC_ = n_cC,_ a_t_ = −a_c_, b_t_ = −b_c_, a_u_ = 0, b_u_ = 0, Y_tT_ = Y_cT_ and Y_tC_ = Y_cC_, where this scenario can be realized when underlying true means are independent of guess (so that biases are introduced only via a's and b's); no bias among subjects who answered “Don't know”; and among those who provided treatment guesses, biases (due to over vs. under-reporting) cancel out *within* each arm. This situation can be regarded as another plausible form of effective blinding which yields a combination of various balances within each arm; see the ‘Random guess’ scenario below.

Next, under n_iT_ = n_iC_ = n_i,_ the relative estimate of the treatment effect is unbiased:

$$\left[\sum_{i}\left(n_{iT}Y_{iT}^{\prime }\right)/\sum_{i}n_{iT}\right]÷\left[\sum_{i}\left(n_{iC}Y_{iC}^{\prime }\right)/\sum_{i}n_{iC}\right]=\left[\sum_{i}\left(n_{iT}Y_{iT}\right)/\sum_{i}n_{iT}\right]÷\left[\sum_{i}\left(n_{iC}Y_{iC}\right)/\sum_{i}n_{iC}\right]$$

if, but not only if, in every stratum, either (1) a_i_ = 0 and (1a) all b_i_'s are equal or (1b) all Y_iC_'s are equal and all Y_iT_'s are equal; or (2) Y_iC_ = Y_iT_. Moreover, when n_iT_ = n_iC_ = n_i_ may or may not be true, if the same 4 conditions as in (3) above are met, unbiased estimation is achieved. Finally, the unbiasedness of OR is even rarer, with equality holding if, but not only if, either (1) Y_iC_ = Y_iT_ or (2) a_i_ = 0 and b_i_ = 0, but unlikely otherwise. Yet, under some conditions, say, the rare disease assumption (e.g., <10%), RR and OR would be close [10], [11].

Now, we introduce different blinding scenarios through representative guess status in (Treatment, Control) = (random, random), (correct, opposite), (correct, random), and (correct, correct), where we will call these four classifications ‘Random guess’, ‘Wishful thinking’, ‘Unblinded in one arm’, and ‘Unblinded in both arms’, respectively, for convenience. Nine blinding scenarios may be classified based on the proportion of correct guesses, and these four scenarios have been shown to be relatively common in systematic reviews [12], [13], [14] and are covered in this study; extensions to the remaining five scenarios are straightforward. For example, ‘Random guess’ may correspond mathematically to blinding index (BI) values of (0, 0), ‘Wishful thinking’ to (k, −k), ‘Unblinded in one arm’ to (k, 0), and ‘Unblinded in both arms’ to (k, k), with a positive proportion of k, where BIs for treatment arm and control arm are defined as:

$${\text{BI}}_{\text{T}}=\left(2*{\text{n}}_{\text{tT}}/\left({\text{n}}_{\text{tT}}+{\text{n}}_{\text{cT}}\right)-1\right)*\left({\text{n}}_{\text{tT}}+{\text{n}}_{\text{cT}}\right)/\left({\text{n}}_{\text{tT}}+{\text{n}}_{\text{uT}}+{\text{n}}_{\text{cT}}\right)\text{,}$$

$${\text{BI}}_{\text{C}}=\left(2*{\text{n}}_{\text{cC}}/\left({\text{n}}_{\text{tC}}+{\text{n}}_{\text{cC}}\right)-1\right)*\left({\text{n}}_{\text{tC}}+{\text{n}}_{\text{cC}}\right)/\left({\text{n}}_{\text{tC}}+{\text{n}}_{\text{uC}}+{\text{n}}_{\text{cC}}\right)\text{.}$$

BI may serve as an indicator of potential unblinding through quantifying ‘imbalance’ between the two statuses of the identified guesses, i.e., T vs. C [15]. Here, we chose Bang et al.’s BI because it is widely used in practice, including in meta-analyses, and it assesses blinding separately for different arms (unlike James et al.'s BI that provides one value) so that it could capture different blinding patterns in different arms [12], [13], [14], [16], [17], [18], [19]. Roughly speaking, BI = 0 means that the proportions of correct and incorrect guesses are equal, adjusting for the count of “Don't know”. Here, k = 20% has been used as an ad-hoc threshold for classification purposes [14], [15], [20]. Note that the condition, n_iT_ = n_iC_ = n_i_, imposed in Mathieu et al. implies BI_T_ = −BI_C_. Hence, in practice, if BI_T_ ≈ −BI_C_ ≫ 0, say, >20%, we may designate as the ‘Wishful thinking’ scenario. In contrast, if a set of the conditions in (3) above are satisfied (e.g., n_tT_ = n_cT_, n_tC_ = n_cC_), so BI_T_ ≈ −BI_C_ ≈ 0, we may designate as the ‘Random guess’ scenario. These two scenarios may constitute effective blinding.

Although these theoretical conditions identified in simplistic models/settings are critical in the improvement of our understanding about blinding and its potential impact on treatment effect, it is not straightforward to understand the extent of bias in any given trial. Exact cancellations would be nearly impossible and some conditions are too restrictive or highly implausible (for example, b_i_ = b), so that bias is highly likely in most cases, especially, in more general or realistic settings we simulated below.

## 3 Simulation study

### 3.1 Configuration and data generation

In this section, we examine the empirical bias in the treatment effect by simulation in the combinations of: 1) outcomes (continuous and binary); 2) parameters (mean difference/RD, mean ratio/RR, and OR); 3) hypotheses (null and non-null effects); and 4) different blinding scenarios. In brief, we followed Mathieu et al.'s setting/framework for data presentation and generation as in Table 1. We first generated true outcomes and then divided into 3 subgroups based on guess status. After that, we did add or subtract random bias in 2 guess groups, except for the “Don't know” group. Sample size of each subgroup determines blinding index and different blinding scenarios.

For continuous outcome, we defined the relationship between observed and true outcomes with bias factors, a's and b's:

$$Y_{ijl}^{\prime }=Y_{jl}+a_{il}+b_{il}Y_{jl}$$

where i = t, u, c; j = T, C; and l indexes subjects within each stratum defined by i and j. Thus, Y, a and b all vary over subjects (with subscript l), and the underlying true outcomes are assumed to be independent of guess (no subscript i), so that the observed outcome depends on guess i through *a*_*il*_ and *b*_*il*_. For binary outcome, we assumed the relationship between the true and observed outcomes with the bias factors, *a*_*il*_ and *b*_*il*_, in the logit scale:

$$\text{logit}\left[P\left(Y_{ijl}=1\right)\right]=\text{α}+\text{β}*\text{I}\left(\text{j}=\text{T}\right)\;$$

$$\text{logit}\left[P\left(Y_{ijl}^{\prime }=1\right)\right]=\text{α}+\text{β}*\text{I}\left(\text{j}=\text{T}\right)+a_{il}+b_{il}Y_{jl}\text{.}$$

In all simulations, we generated data under the null (i.e., 0 in difference and 1 in ratio) and non-null treatment effects. For continuous outcome, we generated true outcome data from a normal distribution with the variance of 1 and the difference in the means of 0 (=1−1 for null effect) and 0.3 (=1.3−1 for non-null effect) so the corresponding ratios in the mean of 1 and 1.3. Simulation configurations along with results are described in Table 2. For binary outcome, we simulated data when OR of exp (0) = 1 (under the null) and exp (0.8) = 2.23 (under the non-null) for treatment effect parameter, and the results are presented in Table 3.

**Table 2:** Simulation for continuous outcome.

Treatment effect	Belief status	Blinding scenario	Bias in mean difference		Bias in mean ratio	
			Balance			
			Yes	No	Yes	No
Null	Baseline	Random guess	0	0	0	0
		Wishful thinking	0	0	0	1
		Unblinded-1 arm	8	13	7	12
		Unblinded-2 arms	16	24	16	24
Null	Depends on outcome	Random guess	0	0	0	0
		Wishful thinking	0	0	0	0
		Unblinded-1 arm	9	14	8	13
		Unblinded-2 arms	16	24	17	24
Null	Both	Random guess	0	0	0	0
		Wishful thinking	0	0	1	1
		Unblinded-1 arm	16	27	16	24
		Unblinded-2 arms	32	47	36	49
Non-null	Baseline	Random guess	0	0	0	−2
		Wishful thinking	0	0	−2	−4
		Unblinded-1 arm	8	13	7	10
		Unblinded-2 arms	16	24	19	25
Non-null	Depends on outcome	Random guess	0	2	1	1
		Wishful thinking	2	6	0	1
		Unblinded-1 arm	11	20	11	17
		Unblinded-2 arms	18	30	21	31
Non-null	Both	Random guess	0	2	0	−1
		Wishful thinking	3	7	−1	−3
		Unblinded-1 arm	19	33	18	25
		Unblinded-2 arms	34	54	43	58

N = 500 subjects per arm. 500 simulations were performed.

Bias = observed-truth, adjusted for sampling bias and multiplied by 100.

**Table 3:** Simulation for binary outcome.

Treatment effect	Belief status	Blinding scenario	Bias in risk difference		Bias in risk ratio		Bias in odds ratio	
			Balance					
			Yes	No	Yes	No	Yes	No
Null	Baseline	Random guess	0	0	0	−1	0	−1
		Wishful thinking	0	0	−1	−1	−1	−1
		Unblinded-1 arm	1	1	7	13	8	15
		Unblinded-2 arms	1	2	15	26	16	29
Null	Depends on outcome	Random guess	0	0	0	0	0	0
		Wishful thinking	0	0	0	0	0	0
		Unblinded-1 arm	0	0	1	1	1	1
		Unblinded-2 arms	0	0	2	2	2	3
Null	Both	Random guess	0	0	0	0	0	0
		Wishful thinking	0	0	1	−1	1	−1
		Unblinded-1 arm	1	2	8	15	9	17
		Unblinded-2 arms	2	3	17	30	19	34
Non-null	Baseline	Random guess	0	0	0	−5	0	−4
		Wishful thinking	1	1	−2	−9	0	−5
		Unblinded-1 arm	1	3	13	19	19	32
		Unblinded-2 arms	2	4	30	43	40	63
Non-null	Depends on outcome	Random guess	0	0	0	5	0	7
		Wishful thinking	1	1	5	11	7	16
		Unblinded-1 arm	1	1	7	14	10	20
		Unblinded-2 arms	1	2	9	17	12	25
Non-null	Both	Random guess	0	1	0	1	1	5
		Wishful thinking	1	3	3	3	7	12
		Unblinded-1 arm	2	5	21	33	31	55
		Unblinded-2 arms	3	6	40	63	56	97

N = 500 subjects per arm. 500 simulations were performed.

Bias = observed-truth, adjusted for sampling bias and multiplied by 100.

In general, the magnitude of over and under-reporting of the outcomes (e.g., patient reported pain score) are limited, we independently generated bias factors, *a*_*tl*_ and *b*_*tl*_, from a uniform distribution of U [0, 0.4], and $a_{cl}$ and $b_{cl}$ from a uniform distribution of U [−0.4, 0], while $a_{ul}$ and $b_{ul}$ set to 0. This means that subjects who guess they are in treatment group tend to over-report and those in control group tend to under-report in the balanced manner, say, by 20% under the null with baseline or fixed belief only; we call this ‘Balance = Yes’. In contrast, for the counterpart of ‘Balance = No’, we generated the data with the means of 0.4 and −0.2, in place of 0.2 and −0.2 above, in order to make the magnitude of over-reporting is larger than that of under-reporting, so that cancellations of the bias factors are unlikely. We tried three sub-scenarios regarding belief status: 1) a ≠ 0 but b = 0 (i.e., belief at baseline or fixed belief, independent of outcome); 2) a = 0 but b ≠ 0 (i.e., bias modified by outcome); and 3) a ≠ 0 and b ≠ 0 (i.e., both).

The four blinding scenarios of ‘Random guess’, ‘Wishful thinking’, ‘Unblinded in 1 arm’, and ‘Unblinded in 2 arms’ were implemented through the BIs of (0, 0), (0.4, −0.4), (0.4, 0), (0.4, 0.4), respectively, partly guided by the recent meta-analyses on blinding [12], [13]. Here, 0.4 means that 40% of imbalance in guesses in a given arm, for instance, 60% of subjects guessed treatment, 20% answered ‘Don't know’, and 20% guessed control among subjects in treatment arm, where we denote as 60-20-20%. Since balance in Random guess can be achieved in different manners, we tried the three settings of (1) 33.3-33.3-33.3% for both arms; (2) 40-20-40% for both arms; and (3) 40-20-40% in treatment arm and 33.3-33.3-33.3% in control arm, which all yield BI = 0. We decided to report results from the first setting as results were qualitatively similar.

We generated the data for 500 subjects in each arm. A set of 500 simulation runs were conducted in each combination. We reported the average of sample bias estimates in treatment effect parameter, adjusting for sampling bias (i.e., subtracting the observed bias under the true model); we intentionally used the raw bias so as to closely reflect naturalistic situations, that is, how RCT results are normally reported in practice, and to highlight when unbiasedness (i.e., numerical bias of 0) is achieved. SAS 9.4 (SAS Institute, Cary, NC) was used for data generation and analysis and codes will be available upon request from author. We performed additional analyses as secondary analyses or sensitivity checking: 1) we repeated the entire simulation study with weaker unblinding in one arm or both arms (say, BI = 0.2 in place of 0.4); 2) we tried when true OR < 1 instead of >1; and 3) we tried a model in the prevalence scale, in place of the logit scale, similar to Mathieu et al.'s hypothetical example. We summarized key findings in text below.

### 3.2 Summary of results

Main simulation results are presented in Table 2, Table 3. It is clear overall that Random guess and Wishful thinking scenarios which closely represent successful blinding yielded minimal biases, while Random guess appears to be the most ideal blinding scenario with the estimated bias of ∼0 in most balanced scenarios we tried (Balance = Yes). Bias is non-zero but relatively small in imbalanced settings (Balance = No), where a's and b's do not cancel out. Bias is not always zero under successful blinding, especially, when treatment effect is non-zero, i.e., non-null effect. Small but non-zero bias can occur in the Wishful thinking scenario even when balances in a's and b's are maintained. Under the null effect and successful blinding, bias is shown to be near 0. However, if blinding is unsuccessful, the null effect does not guarantee unbiasedness.

Observed bias tends to be larger for ratio measure, compared to difference measure, and bias could be amplified in OR, compared to RR, when OR lies in 1 to ∞, particularly when OR ≫ RR for non-rare outcome. As theory predicts, unbiasedness is most difficult to be achieved for OR, compared to RD or RR, as numerical cancellations of biases are unlikely. It is also noteworthy that increased unblinding (i.e., two arms rather than one arm, a higher degree of unblinding in a given arm, a ≠ 0 and b ≠ 0) and larger imbalance in the magnitude of over vs. under-reporting can yield larger bias. Thus, we may interpret that balances in guess of T vs. C as well as in over vs. under-reporting lead to more cancellations in biases as theory predicts. Two different scales – prevalence and logit – provide comparable results. When we tried the settings with OR < 1, we reached biases in a lot smaller magnitude. But it is well explained by the range issue, i.e., 0–1 vs. 1−∞.

## 4 Discussion

In this paper, we studied the numerical behaviors of bias in treatment effect when outcomes are affected by subjects' beliefs about treatment they received. Statistical investigation of the potential effect of (un)blinding on treatment effect is highly limited, while anecdotal evidence, concept and intuition have been utilized and reported in clinical communities; blinding must be a good thing, especially double blinding [7], [8], [12]. Mathieu et al. [9] provided a theoretical analysis of simplistic and hypothetical framework that demonstrates effective blinding cannot guarantee unbiasedness in treatment effect, which may yield important implications in RCT practice because most trialists and practitioners regard blinding is an essential component to minimize various biases.

We examined their important findings in a numerical study in realistic and diverse settings, and confirmed their claims are correct. Yet, observed bias tends be small under the original and newly identified settings of effective blinding, so that trialists and readers may not need to substantially change their perception about blinded RCT being the gold standard. We also found that bias estimates can be different in different parameters/settings, and bias can be the largest when blinding is broken – the more severe the unblinding, the larger the bias. We also identified specific contexts where bias is nearly 0. We still believe that the lesson that blinding does not guarantee unbiased treatment effect is important, similarly to the lesson that another norm, the intent-to-treat principle, does not guarantee unbiased estimation under noncompliance [21]. Although bias could heavily depend on the parameter of choice (e.g., RD vs. RR vs. OR; OR > 1 vs. <1), we do not think that parameter should be selected based on the biases we observed/discussed here.

It could be difficult, if not impossible, to control or measure some a's (say, belief before randomization) and b's (e.g., belief developed during trial), but it may be still important to do our best to make bias factors as small as possible or more balanced in opposite directions (e.g., a_t_ ≈ −a_c_, b_t_ ≈ −b_c_), which could be better achieved when patients are more confused between the two interventions under comparison, with the entire team's consorted efforts and novel tricks.

The limitations of our study and discussion points should be noted. First, we employed simple settings and models. We believe that simple settings (in model, parameter, classification, etc.) are preferred as fundamental and general behaviors of the operational characteristics are better elucidated. Particularly, we focused on the allocation ratio of 1:1 as the interpretation of blinding success under other ratios might not be straightforward, say, is the null value 50% or 33% under 1:2? More advanced designs and models have been proposed and may be considered in future [16], [22], [23], [24]. Second, statistically speaking, this problem is a special case with measurement error or misclassification in outcomes [25]. However, we are cautious to recommend statistical correction with hard-to-satisfy-or-verify assumptions partly because of non-statistical reason; more data collection about blinding may do more harm than good [20], [21]. A more desirable approach could be rigorous pilot testing of blinding before actual trial [26], together with better adherence to blinding protocol [5], [20], and stratified analyses based on guess status as exploratory or secondary analysis, similarly to stratified analyses based on compliance status [23], [27], [28]. Third, we used blinding scenario classification with previously used names based on BI for convenience and evaluation purpose. Blinding is a qualitative and empirically unverifiable issue in general, so BI which is a function of the proportions of correct and incorrect guesses can only serve a proxy measure, and different underlying phenomena are possible under the same name. For example, “Wishful thinking” could actually reflect a situation where anything looking like treatment is perceived as real, rather than a well-known psychological tendency to wish to receive a real or better treatment.

In conclusion, we validated Mathieu et al.’s theoretical findings (i.e., bias cannot be eliminated even when perfect blinding is achieved) in our simulations. We also found that observed bias could be relatively small in various realistic settings when blinding is successful in two specific ways or degrees of over vs. under-reporting due to beliefs are comparable, particularly when treatment effect is near null. Thus, clinical implications of theoretical biases on the current RCT practice may not be substantial although it is ideal to remember possible bias, whenever we interpret results from RCTs where blinding could be more problematic. Clinical trial team should do their best to aim at “Random guess” or “Wishful thinking” throughout the trial in terms of blinding. Finally, our study reinforces the old wisdoms, perfect blinding is better than imperfect blinding, and imperfect blinding is better than no blinding, and “balancing” feature is important in RCTs.

## Conflict of interest

None.
